# First record of the cimicomorphan family Plokiophilidae (Hemiptera, Heteroptera) from China, with description of a new species of *Plokiophiloides*

**DOI:** 10.3897/zookeys.1021.56599

**Published:** 2021-03-05

**Authors:** Jiuyang Luo, Yanqiong Peng, Qiang Xie

**Affiliations:** 1 State Key Laboratory of Biocontrol, Sun Yat-sen University, 135 Xingangxi Road, Guangzhou 510275, Guangdong, China Sun Yat-sen University Guangzhou China; 2 Key Laboratory of Tropical Forest Ecology, Xishuangbanna Tropical Botanical Garden, Chinese Academy of Sciences, Kunming 650223, Yunnan, China Xishuangbanna Tropical Botanical Garden, Chinese Academy of Sciences Kunming China

**Keywords:** China, Cimicoidea, Cimicomorpha, new record, new species, Oriental Region, *
Plokiophiloides
*, taxonomy

## Abstract

*Plokiophiloides bannaensis***sp. nov.**, is described from Xishuangbanna, Yunnan Province, representing the first record of the family Plokiophilidae from China. The new species also represents the first record of the genus *Plokiophiloides* in the Oriental Region, a second zoogeographical region besides the Afrotropical Region. Photographs of the live individuals inhabiting a spider web within natural habitats, male and female habitus, wings of adult, male genitalic structures, female abdomen structures and scanning electron micrographs of forewing, head, thorax and legs are provided. A key to all known species of *Plokiophiloides* is presented, with a distribution map.

## Introduction

The family Plokiophilidae China, 1953 is a small group of true bugs, currently containing nine genera and 20 species (including one Baltic amber fossil genus and one fossil species). Their appearance is reminiscent of the Anthocoridae*sensu lato*, ranging in length from 1.2 to 3.0 mm (Schuh and Slater 1995; Popov 2008; Schuh et al. 2015). Most of them were found to inhabit the webs of spiders or embiopterans, so they are also called web-lovers (Schuh and Weirauch 2020).

China and Myers (1929) described the first species of Plokiophilidae from Cuba, naming it *Arachnophila cubana* China & Myers, 1929 and placed it in the family Microphysidae. After more than two decades, China (1953) proposed the new name *Plokiophila* China, 1953 for *Arachnophila* China & Myers, 1929, which was preoccupied by *Arachnophila* Salvadori, 1874 (Aves) and described substitutive new genus and species from Trinidad, *Embiophila myersi* China, 1953 and also established Plokiophilinae as a subfamily of Microphysidae. Subsequently, Carayon (1961) upgraded Plokiophilinae to the family level, which was supported by Štys (1962); Štys (1967) described a new genus and species *Lipokophila chinai* Štys, 1967 from Brazil and provided a key to the Plokiophilidae. Carayon (1974) published a monograph of this family, describing a new genus and seven new species from continental Africa and Brazil. After the 1990s, a series of new genera and species were discovered by Štys (1991), Schuh (in Eberhard et al. 1993), Carpintero and Dellapé (2005), Schuh (2006) and Popov (2008). Recently, Schuh et al. (2015) published an influential monograph on the Plokiophilidae, in which two new genera and three new species were described and a revised higher classification for the family was offered. Mainly based on the male genitalic and female copulatory structures, a new subfamily Heissophilinae and a new tribe Lipokophilini were established by Schuh et al. (2015); in addition, the subfamily Embiophilinae was downgraded to the tribe level. In the present paper, we follow the taxonomic system of Schuh et al. (2015).

The genus *Plokiophiloides* Carayon, 1974 was established by discovery of five new species from the Afrotropical Region. After that, Štys (1991) described a new species *Plokiophiloides steineri* Štys, 1991 from Madagascar and he divided the genus into two species groups *P. asolen* group and *P. tubifer* group, based on the length of the male acus and existence of the external integumental paragenitalia of the female. Štys and Baňař (2016) reported a new free-living species and built a new genus *Neoplokioides*Štys and Baňař 2016, to hold the species; moreover, all the species of *Plokiophiloides tubifer* group were transferred into the genus *Neoplokioides*. At present, the genus *Plokiophiloidess. str.* includes four species, *P. asolen* Carayon, 1974, *P. balachowskyi* Carayon, 1974, *P. pilosus* Carayon, 1974 and *P. steineri* Štys, 1991.

In this work, the new species *Plokiophiloides bannaensis* sp. nov. is described. The family Plokiophilidae is a new record for the fauna of China and the first record of the genus *Plokiophiloides* beyond the Afrotropical Region.

## Materials and methods

Specimens were collected from the webs of wolf spiders *Hippasa* sp. (Araneae: Lycosidae) in low herbaceous plants, in Xishuangbanna Tropical Botanical Garden (XTBG), Chinese Academy of Sciences. The collected specimens were preserved in 80% ethanol.

External structures and genitalic structures were examined by using a Zeiss Discovery V20 stereomicroscope. Measurements (in mm) were taken using the Zeiss Discovery V20 stereomicroscope with ZEN 2.5 pro software. Male genitalia and the female abdomen were macerated in warm 10% potassium hydroxide solution (KOH). Photographs of habitus, forewing, male genitalia and female abdomen were taken using a Canon EOS 7D Mark II camera, equipped with a tube lens and Mitutoyo M Plan Apo 10× objective lens. Scanning electron micrographs of forewing, head, thorax and legs were prepared using a Zeiss EVO MA 10 at the Instrumental Analysis & Research Center of Sun Yat-sen University. Maps were prepared using SimpleMappr (http://www.simplemappr.net/).

Morphological terminology follows Štys (1991), Schuh et al. (2015) and Štys and Baňař (2016). Depository: The type series of *Plokiophiloides bannaensis* sp. nov. is deposited in the Museum of Biology, Sun Yat-sen University, Guangzhou, China (**SYSBM**).

Abbreviations used in the text and figures are as follows:

**a** acus

**ap** articulatory apparatus

**asg** abdominal scent gland orifice

**cf** costal fracture

**cg** corial gland

**cgs** corial glands

**cp** corial process

**Cu** cubitus

**e** egg

**lp** left paramere

**M** media

**ph** phallosoma

**py** pygophore

**R** radius

**rp** right paramere

**Sc** subcostal

**sv** secondary vein

**ts1** first segment of tarsus

**ts2** second segment of tarsus

**t8** abdominal tergite 8

**v8** abdominal ventrite 8

**1An** first anal vein

## Taxonomy

### Family: Plokiophilidae China, 1953

**Subfamily: Plokiophilinae China, 1953**

**Tribe: Plokiophilini China, 1953**

#### Genus: Plokiophiloides

Taxon classificationAnimaliaHemipteraPlokiophilidae

Carayon, 1974

5AEC92E3-E48D-541D-A811-F66EB6864F6E

Plokiophiloides Carayon, 1974: 505.

##### Type species by original designation.

*Plokiophiloides asolen* Carayon, 1974.

##### Comments.

The genus *Plokiophiloides* currently includes four species and can be distinguished from other genera of Plokiophilidae by the following combined characteristics: a) tarsi 2-segmented, b) hemelytron with a distinct cuneus, c) male pygophore tubular, erect, d) fore femora and middle femora without heavy spines on ventral surface, e) posterior margin of pronotum excavated and mesoscutum broadly exposed, f) male acus shorter than pygophore and its basis simple and g) females without paired external paragenitalia.

#### Plokiophiloides bannaensis sp. nov.

Taxon classificationAnimaliaHemipteraPlokiophilidae

F181860F-2CAA-5D88-A663-9E7165CC1C8E

http://zoobank.org/738A74B1-4FC3-4DD1-983A-53A5B0CF59CA

**Figure 1.**
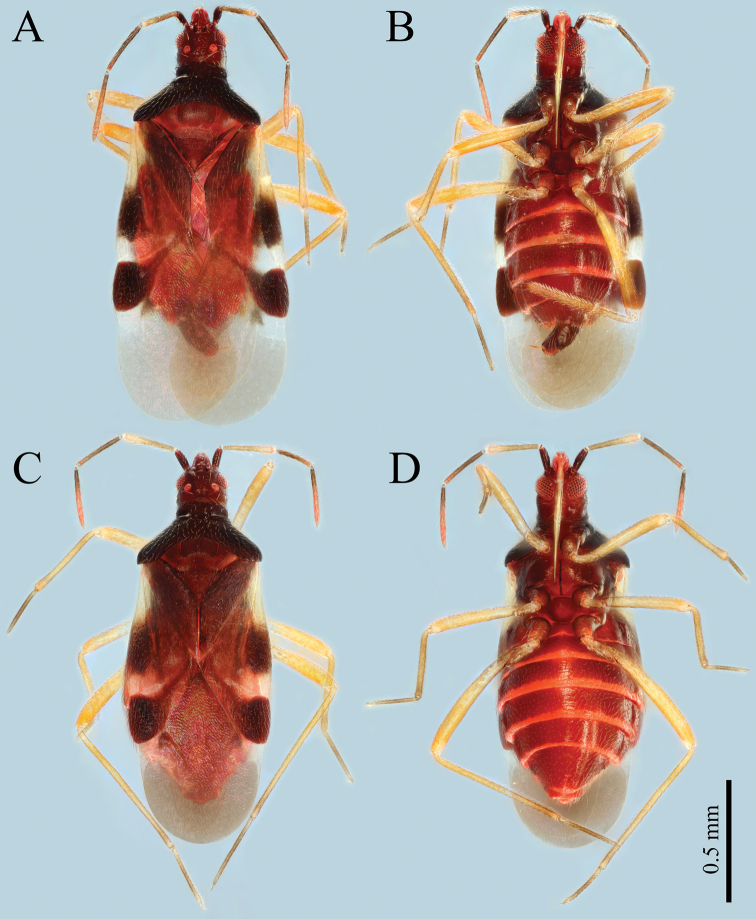
Habitus of *Plokiophiloides bannaensis* sp. nov. **A** male holotype in dorsal view **B** male holotype in ventral view **C** female paratype in dorsal view **D** female paratype in ventral view.

**Figure 2.**
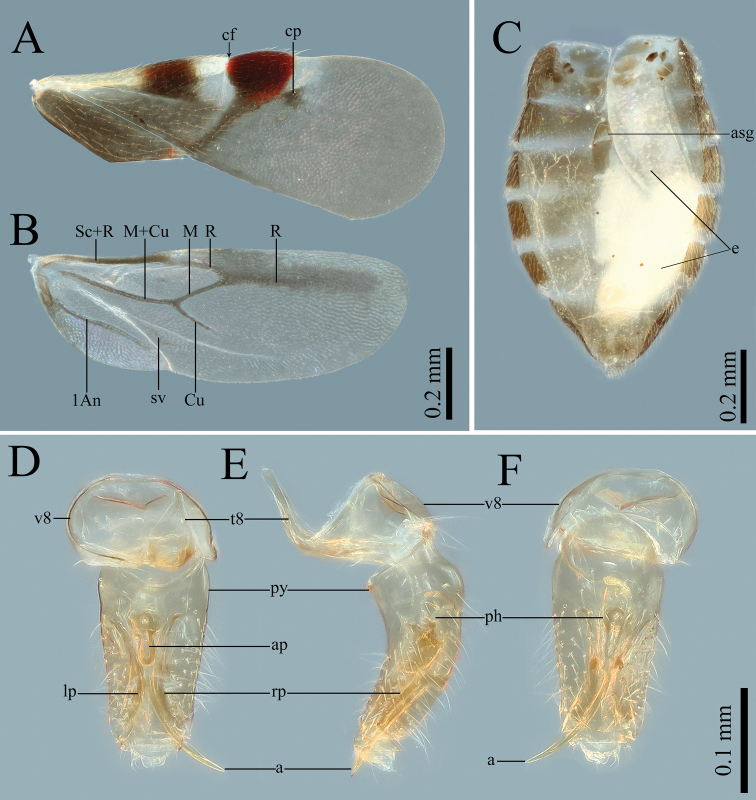
Morphology of *Plokiophiloides bannaensis* sp. nov. **A** male forewing in dorsal view **B** male hind-wing in dorsal view **C** female abdomen in dorsal view **D** male genitalia in dorsal view **E** male genitalia in lateral view **F** male genitalia in ventral view. Abbreviations: a = acus; ap = articulatory apparatus; asg = abdominal scent gland orifice; cf = costal fracture; cp = corial process; e = egg; lp = left paramere; ph = phallosoma; py = pygophore; rp = right paramere; t8 = abdominal tergite 8; v8 = abdominal ventrite 8.

**Figure 3.**
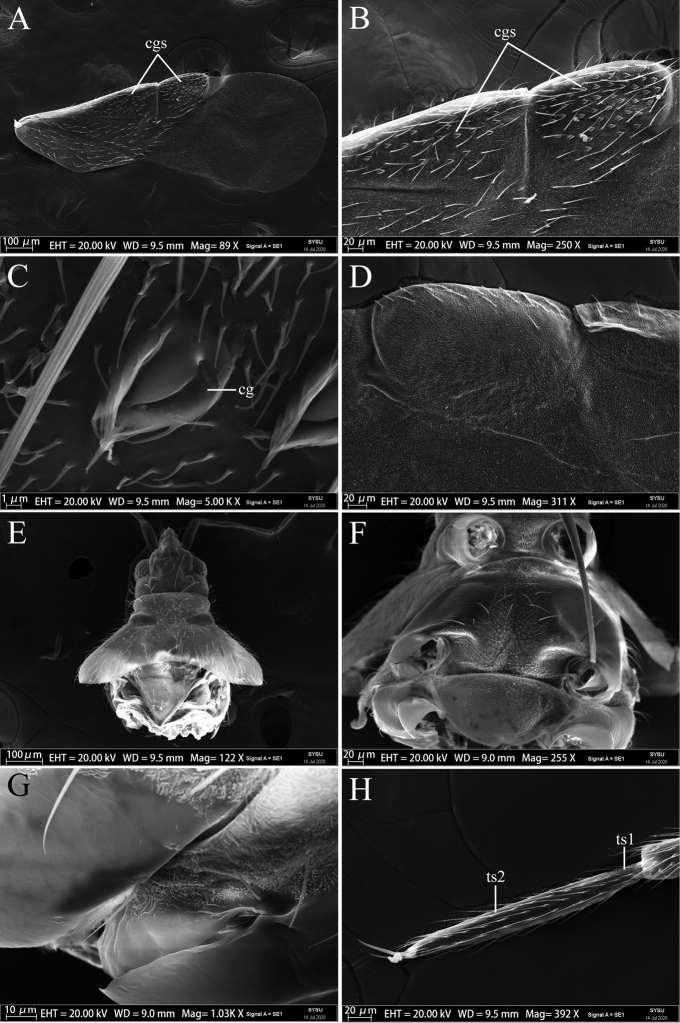
Scanning electron micrographs of *Plokiophiloides bannaensis* sp. nov. **A** male forewing in dorsal view **B** male forewing in dorsal view (with all corial glands) **C** detail of corial gland in dorsal view **D** male cuneus and costal fracture of forewing in ventral view **E** male head and thorax in lateral view (with all wings and legs removed) **F** male thorax in ventral view (with all wings and legs removed) **G** right lateral view of meso- and metathorax **H** male fore tibia anterior view. Abbreviations: cg = corial gland; cgs = corial glands; ts1 = first segment of tarsus; ts2 = second segment of tarsus.

**Figure 4.**
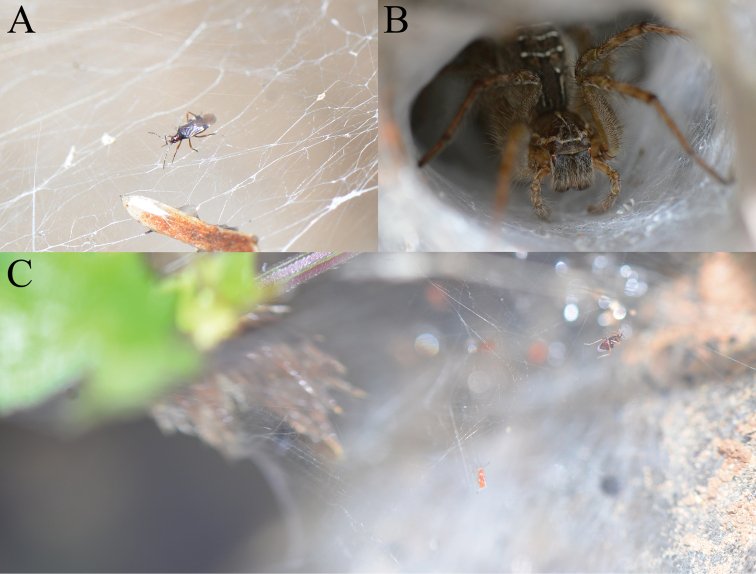
Habitus images of live individuals for *Plokiophiloides bannaensis* sp. nov. and spider host *Hippasa* sp. **A** an adult of *Plokiophiloides bannaensis* sp. nov. on web **B** an individual of *Hippasa* sp. in funnel of the web **C** an adult and a nymph of *Plokiophiloides bannaensis* sp. nov. near funnel of the web.

Figures 1 , 2 , 3 , 4

##### Material.

***Holotype*:** ♂, China, Yunnan Province, Xishuangbanna, Mengla County, Menglun Town, XTBG: 21°54.965'N, 101°16.293'E; ca. 670 m elev.; leg. Qiang Xie & Jiuyang Luo; 2018-VI-27. ***Paratypes***: 5♂♂, 5♀♀, and 2 nymphs, same data as holotype.

##### Diagnosis.

The new species can be distinguished from the congeners by the combined characteristics: body with red pigment; exocorium with ca. 65 corial glands; whitish precuneal spot on forewing conspicuous; hypocostal lamina extending caudally as a short, pale whitish-yellow projection.

##### Description.

**Macropterous male**: Small-sized (1.5–1.7 mm), elongate, relatively flat, forewing exceeding apex of abdomen (Fig. 1A–D). ***Colouration***: Ground colour and head dark reddish-brown, ocelli and ventral surface of head paler. Antennal segment I dark reddish-brown; segment II light brown, distal 1/3 pale yellow, with reddish tinge; segment III brown, distal 1/4 paler, reddish; segment VI brown, distal 2/3 reddish. Labial segments I and II light reddish; segments III and IV yellowish-brown. Pronotum and propleura darker, dark brown to blackish-brown; middle of mesosternum with longitudinal suture, nearly black. Coxae dark reddish-brown; trochanters, femora and tibiae yellowish-brown, sometimes with reddish tinge; tibiae gradually darker to the apices; tarsi brown, basal and distal part paler (Fig. 1A–D). Corium and clavus light brown to brown, middle part of exocorium and outer part of cuneus dark reddish-brown; basal 2/5 of corium light whitish-yellow; the area around costal fracture and the area of membrane that is behind distal end of cuneus whitish. Membrane light brown, with two inconspicuous longitudinal veins; corial process brown (Fig. 2A). Posterior margin of abdominal sterna nearly red.

***Surface and vestiture*:** Head, thorax, corium and clavus forewing and abdomen covered with relatively sparse and uniformly long semi-erect setae (Fig. 1A–D). Thorax (except for calli, mesoepleurae and metapleurae) and surface of forewing densely covered by microtrichia (Fig. 3B–G). Antennae and legs covered with dense, long, semi-erect setae.

***Structure*: Head** porrect, cylindrical, length subequal to width. Eyes away from collar; minimum dorsal interocular distance greater than 2× the same distance ventrally. Ocelli large, widely separated from each other. Two pairs of strikingly-long setae placed on dorsal surface of head, one pair of setae located in inner part of eyes, at level of anterior margin of eyes, the other located in the posterolateral part of ocelli (Fig. 3E). Antennae thin, terete; segment I thicker than the others, segment II gradually thickening towards the apex, tip of segment IV fusiform; ratio of length of antennal segments I : II : III : IV = 3 : 7 : 7 : 9 (see Table 1). Labium slender, nearly reaching to posterior margin of mesosternum, segment I very short and wide; ratio of length of labial segments I : II : III : IV = 1 : 2.3 : 4 : 7. **Thorax**: pronotum trapezoidal, with distinct collar, one pair of extraordinarily-long setae placed in dorsal surface of collar; lateral margins of posterior lobe straight; posterior margin strongly excavated, broadly exposed mesoscutum; ratio of maximum length to maximum width = 1 : 2 (Fig. 3E–G). Costal margin of corium almost straight; hypocostal lamina extending caudally as a short, pale whitish-yellow end; costal fracture deep, almost reaching medial furrow, located ca. 3/4 from the base of corium (Fig. 3A, B). Venation of hind-wing as in Fig. 2B. Legs slender, femora and tibiae without heavy spines on distoventral surface, fore- and middle tibiae with a cleaning comb on apices of ventral surface; tarsi long and slender, 2-segmented, segment I very short, segment II elongate; claws long, inner claw longer than outer (Fig. 3H). **Abdomen**: Sterna entire, mediotergites membranous, except for segments I and II with discontinuous remnants of mediotergites and segment VII with a sclerotised medial plate. Abdominal scent gland orifice distinct. Segment VIII with a long, bell-shaped tergum and a U-shaped ventrite. **Male genitalia**: Pygophore tubular, symmetrical, slightly curved, ventral basal part moderately rounded and bulging. Parameres symmetrical. Phallosoma simple, long. Acus thin, shorter than pygophore, generally curving to the right (Fig. 2D–F).

**Table 1.** Measurements (in mm) of *Plokiophiloides bannaensis* sp. nov.

Body part	Male holotype	Male (n = 5)	Female (n = 5)	Last instar nymph (n = 2)
length of body	1.62	1.50–1.71	1.59–1.68	1.34–1.37
length of head	0.22	0.19–0.21	0.23–0.25	0.20
greatest width across eyes	0.21	0.19–0.23	0.19–0.22	0.20–0.21
minimum dorsal interocular distance	0.14	0.12–0.14	0.12	0.14–0.15
minimum ventral interocular distance	0.05	0.05	0.05	0.09–0.12
total length of antennae	0.76	0.72–0.83	0.74–0.80	0.67–0.70
length of antennal segment I	0.09	0.07–0.09	0.08–0.09	0.08–0.09
length of antennal segment II	0.2	0.20–0.23	0.19–0.22	0.17
length of antennal segment III	0.21	0.18–0.23	0.19–0.23	0.17–0.18
length of antennal segment IV	0.26	0.26–0.28	0.26–0.28	0.24–0.27
total length of labium	0.55	0.55–0.59	0.53–0.58	0.48–0.54
length of labial segment I	0.04	0.03–0.05	0.03–0.05	0.03
length of labial segment II	0.08	0.09–0.10	0.08–0.10	0.09–0.10
length of labial segment III	0.15	0.15–0.17	0.15–0.17	0.12–0.15
length of labial segment IV	0.28	0.26–0.28	0.25–0.28	0.24–0.26
greatest length of pronotum	0.26	0.23–0.27	0.24–0.26	0.18–0.19
width of pronotum	0.48	0.45–0.53	0.44–0.50	0.28–0.30
length of fore femur	0.34	0.31–0.38	0.31–0.33	0.29–0.30
length of fore tibia	0.34	0.33–0.40	0.34–0.35	0.31–0.33
length of middle femur	0.38	0.34–0.40	0.36–0.38	0.34–0.36
length of middle tibia	0.36	0.33–0.42	0.37–0.40	0.33–0.34
length of hind femur	0.49	0.49–0.55	0.50–0.53	0.45–0.47
length of hind tibia	0.56	0.56–0.64	0.56–0.60	0.51
length of forewing	1.08	1.05–1.24	1.07–1.19	
length of abdomen	0.59	0.54–0.76	0.73–0.91	0.63–0.65
width of abdomen	0.45	0.36–0.57	0.54–0.57	0.41–0.43

***Macropterous female*: Colouration, surface and vestiture and structures of head and thorax** as in male. **Abdomen**: Pregenital segments as in male, except for segment VII-IX with sclerotised, medial plates (Fig. 2C). **Female genitalia**: No paragenitalia present, boundary of ventrites VIII and IX are unidentified; proctiger simple.

##### Etymology.

The specific name is derived from the abbreviated form of Xishuangbanna, the type locality of the new species.

##### Distribution.

**Figure 5.**
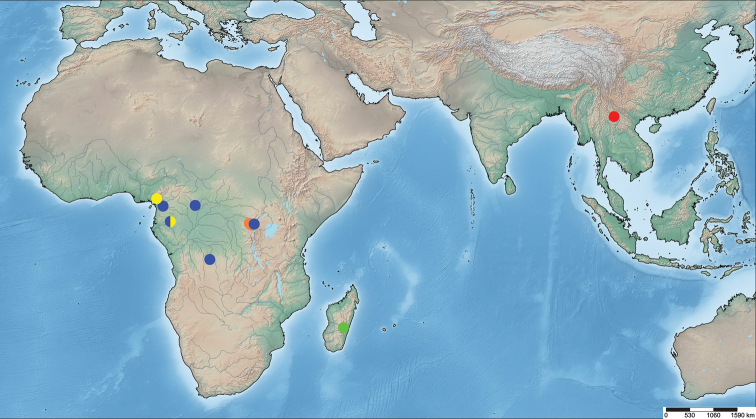
Distribution of all species of *Plokiophiloides* Carayon, 1974, red dot = type locality of *P. bannaensis* sp. nov.; blue dots = *P. asolen*; green dot = *P. steineri*, orange dots = *P. pilosus*, yellow dots = *P. balachowskyi*.

Known only from the type locality, XTBG, Xishuangbanna, Yunnan Province, China (Fig. 5).

#### Key to the species of *Plokiophiloides* Carayon, 1974

Based partly on Carayon (1974), Štys (1991) and Štys and Baňař (2016)

**Table:** 

1	Hypocostal lamina on ventral surface of forewing extending caudally as a long, dark carina	**2**
–	Hypocostal lamina on ventral surface of forewing not extending in a carina	**3**
2	Proximal 1/2 of excorium devoid of corial glands; altogether 18–25 corial glands on forewing. Whitish precuneal spot inconspicuous. Body length 1.2–1.6 mm. (Tropical West Africa)	***P. balachowskyi* Carayon, 1974**
–	Only proximal 1/3 of excorium lacking corial glands; altogether almost 50 corial glands on forewing. Whitish precuneal spot inconspicuous. Body length 2.0–2.6 mm. (Madagascar)	***P. steineri* Štys, 1991**
3	Body without red pigment. Large basal membrane cell distinct. Exocorium with corial glands occurring almost up to its base; altogether ca. 60 corial glands on forewing. Body length 1.3–2.0 mm. (Tropical West and Central Africa)	***P. asolen* Carayon, 1974**
–	Body with red pigment. Membrane cell indistinct or absent	**4**
4	Membrane cell absent. Only the very distal part of exocorium with corial glands; altogether ca. 30 corial glands. Macropterous or brachypterous. Body length 1.3–1.6 mm (Tropical Central Africa)	***P. pilosus* Carayon, 1974**
–	Membrane cell indistinct. The distal 1/4 and middle part of exocorium with two groups of corial glands; altogether ca. 65 corial glands. Macropterous. Body length 1.5–1.7 mm (Tropical Asia: China)	***P. bannaensis* sp. nov.**

## Discussion

Lifestyle of all species of Plokiophilidae is reviewed in Table 2. Amongst them, only two species of the genus *Embiophila* were directly discovered in symbiosis with Embiodea. In addition to *Plokiophiloides bannaensis* sp. nov. (Fig. 4A–C), another eleven species belonging to seven genera were directly discovered in symbiosis with Araneae. Only one species *Neoplokiodes raunoi* was confirmed to be not associated with webs of spiders or embiids by observations during several expeditions (Štys and Baňař 2016). According to our field observation, individuals of *Plokiophiloides bannaensis* sp. nov. were found on five spider webs and there were about 5–8 individuals of them on each web. We examined about 30 specimens, including adults and last-instar nymphs, but other instar nymphs and eggs were not found. Adults and last-instar nymphs of web-lovers like to gather near the entrance of the funnel part of webs, which may be convenient for feeding on the remains of the prey of spiders. The new species of Plokiophilidae and its host spider *Hippasa* sp. are currently only found in tropical China.

**Table 2.** Distribution (using 2-letter country codes) of all known species of Plokiophilidae, lifestyle and symbiotic relationships amongst them and their hosts.

Species of plokiophilids	Distribution	Lifestyle	Species of host	Reference
*Embiophila africana* Carayon, 1974	CG	symbiosis	Embiodea: Embiidae: “*Dihybocercus femorata* (Navas)”	Carayon 1974
*Embiophila maesi* Carpintero & Dellapé, 2005	NI	unknown	unknown, the specimens were collected with Malaise traps	Carpintero and Dellapé 2005
*Embiophila myersi* China, 1953	TT	symbiosis	Embiodea: an unidentified embiid species	China 1953
*Heissophila macrotheleae* Schuh, 2006	TH, ID	symbiosis	Araneae: Hexathelidae: *Macrothele* sp.	Schuh 2006; Schuh et al. 2015
*Lipokophila chinai* Štys, 1967	BR	unknown	unknown, the specimens were collected in litter	Štys 1967
*Lipokophila eberhardi* Schuh, 1993	CR, PA	symbiosis	Araneae: Zoropsidae: *Tengella radiata* (Kulczyński, 1909)	Eberhard et al. 1993; Cambra et al. 2014
*Lipokophila stysi* Carayon, 1974	BR	unknown	unknown	Carayon 1974
*Lipokophila tengella* Schuh, 1993	CR	symbiosis	Araneae: Zoropsidae: *Tengella radiata* (Kulczyński, 1909)	Eberhard et al. 1993
*Monteithophila fijiensis* Schuh et al., 2015	FJ	unknown	unknown	Schuh et al. 2015
*Monteithophila queenslandana* Schuh et al., 2015	AU	symbiosis	Araneae: an unidentified spider species	Schuh et al. 2015
*Neoplokioides biforis* (Carayon, 1974)	GA	symbiosis	Araneae: Dipluridae: *Lathrothele catamita* (Simon, 1907)	Carayon 1974; Štys and Baňař 2016
*Neoplokioides raunoi* Štys & Baňař, 2016	MG	free living	unknown, the specimens were collected in leaf litters	Štys and Baňař 2016
*Neoplokioides tubifer* (Carayon, 1974)	KE	symbiosis	Araneae: an unidentified spider species	Carayon 1974
*Paraplokiophiloides schwendingeri*Schuh et al., 2015	TH	symbiosis	Araneae: Hexathelidae: *Macrothele* sp.	Schuh et al. 2015
† *Pavlostysia wunderlichi* Popov, 2008	Baltic amber.	unknown	Unknown, no possible hosts were reported in the same amber	Popov 2008
*Plokiophila cubana* (China & Myers, 1929)	CU	symbiosis	Araneae: Dipluridae: *Diplura macrura* (Koch, 1841)	China and Myers 1929
*Plokiophiloides asolen* Carayon, 1974	AO, CF, CG, CM, GA,	symbiosis	Araneae: Agelenidae: *Agelena consociata* Denis, 1965	Carayon 1974
*Plokiophiloides balachowskyi* Carayon, 1974	CM, GA	symbiosis	Araneae: Agelenidae: *Agelena republicana* Darchen, 1967	Carayon 1974
*Plokiophiloides bannaensis* sp. nov.	CN	symbiosis	Araneae: Lycosidae: *Hippasa* sp.	present paper
*Plokiophiloides pilosus* Carayon, 1974	CG	unknown	unknown, the specimens were collected in soil samples	Carayon 1974
*Plokiophiloides steineri* Štys, 1991	MG	unknown	unknown, the specimens were collected in flight interception traps	Štys 1991

Notes. Country codes of countries: AO = Angola, AU = Australia, BR = Brazil, CF = Central African Republic, CG = Congo, CM = Cameroon, CN = China, CR = Costa Rica, CU = Cuba, FJ = Fiji, GA = Gabon, ID = Indonesia, KE = Kenya, MG = Madagascar, NI = Nicaragua, TH = Thailand, TT = Trinidad and Tobago. The symbol † indicates fossil species.

The geographic distribution pattern of *Plokiophiloides* is shown in Fig. 5. *Plokiophiloides bannaensis* sp. nov. was found in the Oriental Region and the remaining four species in the Afrotropical Region. Such an unusual intermittent distribution of *Plokiophiloides* reminds us that tropical regions between the Afrotropical and Oriental Regions may contain a high diversity of hidden species in this genus. Similarly, three species of *Embiophila* are separately distributed in the Neotropical and Afrotropical Regions. Therefore, more investigation is needed to clarify the species diversity and thus the distribution pattern of Plokiophilidae in pantropical regions.

## Supplementary Material

XML Treatment for Plokiophiloides

XML Treatment for Plokiophiloides bannaensis
